# Genome-wide SNP identification and QTL mapping for black rot resistance in cabbage

**DOI:** 10.1186/s12870-015-0424-6

**Published:** 2015-02-03

**Authors:** Jonghoon Lee, Nur Kholilatul Izzah, Murukarthick Jayakodi, Sampath Perumal, Ho Jun Joh, Hyeon Ju Lee, Sang-Choon Lee, Jee Young Park, Ki-Woung Yang, Il-Sup Nou, Joodeok Seo, Jaeheung Yoo, Youngdeok Suh, Kyounggu Ahn, Ji Hyun Lee, Gyung Ja Choi, Yeisoo Yu, Heebal Kim, Tae-Jin Yang

**Affiliations:** Department of Plant Science, Plant Genomics and Breeding Institute, and Research Institute of Agriculture and Life Sciences, College of Agriculture and Life Sciences, Seoul National University, Seoul, 151-921 Republic of Korea; Indonesian Research Institute for Industrial and Beverage Crops (IRIIBC), Pakuwon, Sukabumi, Indonesia; Department of Horticulture, Sunchon National University, Suncheon, 540-950 Republic of Korea; Joeun Seed, #174, Munbang-Ri, Cheonhan-Myun, 367-833 Goesan-Gu, Chungcheongbuk-Do Korea; Research Center for Biobased Chemistry, Korea Research Institute of Chemical Technology, Daejeon, 305-600 Yusong-Gu Republic of Korea; Arizona Genomics Institute, School of Plant Sciences, University of Arizona, Tucson, Arizona 85721 USA; Department of Agricultural Biotechnology, Seoul National University, Seoul, 151-921 Republic of Korea; CHO & KIM genomics, Seoul National University Mt.4-2, Main Bldg. #514, SNU Research Park, NakSeoungDae, Seoul, 151-919 Gwanakgu Republic of Korea

**Keywords:** Cabbage, Whole-genome resequencing, Genetic linkage map, Black rot, QTL

## Abstract

**Background:**

Black rot is a destructive bacterial disease causing large yield and quality losses in *Brassica oleracea*. To detect quantitative trait loci (QTL) for black rot resistance, we performed whole-genome resequencing of two cabbage parental lines and genome-wide SNP identification using the recently published *B. oleracea* genome sequences as reference.

**Results:**

Approximately 11.5 Gb of sequencing data was produced from each parental line. Reference genome-guided mapping and SNP calling revealed 674,521 SNPs between the two cabbage lines, with an average of one SNP per 662.5 bp. Among 167 dCAPS markers derived from candidate SNPs, 117 (70.1%) were validated as bona fide SNPs showing polymorphism between the parental lines. We then improved the resolution of a previous genetic map by adding 103 markers including 87 SNP-based dCAPS markers. The new map composed of 368 markers and covers 1467.3 cM with an average interval of 3.88 cM between adjacent markers. We evaluated black rot resistance in the mapping population in three independent inoculation tests using F_2:3_ progenies and identified one major QTL and three minor QTLs.

**Conclusion:**

We report successful utilization of whole-genome resequencing for large-scale SNP identification and development of molecular markers for genetic map construction. In addition, we identified novel QTLs for black rot resistance. The high-density genetic map will promote QTL analysis for other important agricultural traits and marker-assisted breeding of *B. oleracea*.

**Electronic supplementary material:**

The online version of this article (doi:10.1186/s12870-015-0424-6) contains supplementary material, which is available to authorized users.

## Background

Cabbage *(Brassica oleracea* L.) is one of the most important vegetable crops, and is consumed as a food worldwide due to its healthy compounds for humans. Besides its economic importance, cabbage is considered a valuable plant for the study of genome evolution because it contains a CC genome, which represents one of three basic diploid *Brassica* species in the U’s triangle [1]. Recently, two draft genome sequences of *B. oleracea* were reported [2,3], and the availability of this reference genome enhances our understanding of the genome architecture of *B. oleracea* and the evolution of *Brassica* species, as well as facilitates identification of genes associated with important traits for breeding.

Black rot is one of the most devastating diseases to crucifers including *B. oleracea* and is caused by the vascular bacterium *Xanthomonas campestris* pv. *campestris* (Pammel) Dowson (*Xcc*). The disease infects the host plants through hydathodes, wounded tissue, insects and stomata [4,5]. The main disease symptoms are V-shaped chlorotic lesions at the margins of leaves, necrosis and darkening of leaf veins, which lead to serious production losses in vegetable crops [6]. Accordingly, development of cultivars resistant to black rot has been a priority for breeders.

Several methods have been attempted to control black rot disease, including crop diversification and rotation, production of disease-free seed, pre-treatment of seed with bactericide, elimination of potential pathogen sources such as infected crop debris and weeds, and planting of resistant cultivars [7]. Among these, utilization of resistant cultivars is one of the most effective and efficient ways to reduce disease incidence and crop loss. However, the development of commercially acceptable resistant varieties has proven to be extremely difficult due to the lack of studies on genetics and breeding for resistance in cabbage. Two major factors hinder black rot resistance breeding in *B. oleracea*: multigenic control of resistance and emergence of new races of the pathogen that overcome host resistance [8]. Nine races of *Xcc* have been identified [9], among which races 1 and 4 are the major pathogens causing black rot disease in *B. oleracea* crops [10]. Therefore, obtaining *B. oleracea* cultivars that have resistance to both races is considered a prerequisite to control black rot disease [11].

Molecular markers are highly useful for genomic analysis and allow exploration of heritable traits and the corresponding genomic variation [12]. DNA markers are now key components of crop improvement programs, and are applied to identify cultivars, analyze genetic diversity, construct linkage maps and identify quantitative trait loci (QTL) [13]. Advances in molecular markers have facilitated the identification of interesting traits via marker-assisted selection (MAS) in plant improvement. Marker-based approaches represent an effective and rapid strategy for identifying and transferring useful genes in breeding programs [14]. Furthermore, the identification of markers linked to QTL can allow analysis of the consistency of QTL effects across different environments and genetic backgrounds, and increase the frequency of favorable alleles during selection [15]. Several QTLs for black rot resistance in *B. oleracea* have been reported, including two on linkage groups 1 and 9, and two additional QTLs on linkage group 2 [15], as well as two other major QTLs on linkage groups 2 and 9, and two minor QTLs on linkage groups 3 and 7 [16]. Moreover, three QTLs analyzed using SNP markers in the F_2_ mapping population derived from a cross between resistant cabbage and susceptible broccoli were found on linkage groups 2, 4 and 5, and exhibited significant effects in black rot resistance [4]. Recently, three further QTLs for black rot resistance were also detected in linkage groups 5, 8 and 9 [5]. In total, 14 QTLs with major and minor effects have been mapped on eight different *B. oleracea* chromosomes, suggesting that resistance to black rot disease is complex and quantitatively controlled by multiple genes in *B. oleracea*.

Successful QTL mapping requires a large number of genetic markers [17]. Markers based on simple sequence repeats (SSRs) and single nucleotide polymorphisms (SNPs) are commonly used due to their advantages over other types of genetic markers. SSR markers are highly reproducible, highly polymorphic, and amenable to automation. However, next-generation sequencing (NGS) technology makes SNP markers preferable to SSR markers [18]. SNPs have proved to be universal as well as the most abundant forms of genetic variation even among individuals of the same species [19]. Therefore, SNP markers exhibit higher polymorphism than SSR markers [20,21].

In this study, we have resequenced two parental cabbage lines up to 20× genome coverage and conducted a genome-wide survey for SNPs. We validated the SNPs and developed derived cleaved amplified polymorphic sequences (dCAPS) markers for resistance against black rot disease. The genome-wide catalog of SNPs, the high-density map derived from a mapping population generated from elite cabbage breeding lines with a narrow genetic background, and the QTLs reported herein all will be valuable for both breeding and genetic research in *B. oleracea*.

## Results

### Whole-genome resequencing of two cabbage parental lines and SNP detection

Whole genome sequencing data included about 114 million raw reads for C1184 and 113 million for C1234 (Table 1). The recently assembled *B. oleracea* genome sequence consists of 488.6 Mb, including 446.9 Mb in 9 pseudo-chromosomes and 41.2 Mb of unanchored scaffolds, and corresponding to almost 75% of the estimated genome size (648 Mb) [3]. Our new sequencing data represented approximately 18-fold genome coverage for both parental lines based on the estimated genome size. We mapped each set of paired reads onto the nine pseudo-chromosomes of reference genome sequence. In total, almost 94 million raw reads (82.1%) and 88 million (77.6%) from C1184 and C1234, respectively, were successfully aligned to the reference genome. The average mapping depth was 21.2- and 20-fold for C1184 and C1234, respectively.

**Table 1:** **Summary of whole-genome resequencing data for** ***B. oleracea*** **lines**

	**C1184**	**C1234**
**Raw reads**	**114,454,524**	**113,830,992**
**Raw bases**	**11,559,906,924**	**11,496,930,192**
**Coverage of** ***B.oleracea*** **genome**	**17.8 ×**	**17.7 ×**
**GC (%)**	**36.1**	**35.6**
**Mapped reads**	**93,956,750**	**88,382,752**
**Mapped percentage (%)**	**82.1**	**77.6**
**Mapped bases**	**9,489,631,750**	**8,926,657,952**
**Mapping depth (average)**	**21.2**	**20.0**

The total number of SNPs relative to the reference sequence and average SNP densities were very similar in both parental lines. Approximately 1.20 and 1.24 million high-quality SNPs are identified in C1184 and C1234, respectively, by comparison to the reference genome. On average, a SNP was detected in each 372.8 bp in C1184, and each 360.0 bp in C1234. Chromosome C03 of both lines had the most SNPs, whereas the fewest SNPs were found on chromosome C06 of C1184 and chromosome C04 of C1234.

**Figure 1:**
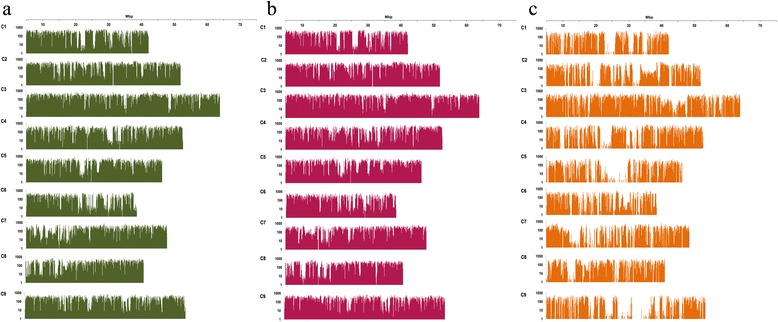
**Distribution of SNPs in the pseudo-chromosomes of** ***B. oleracea.*** SNPs within 100-kb intervals are shown for **(a)** Reference vs. C1184; **(b)** Reference vs. C1234; **(c)** C1184 vs. C1234.

These SNPs were merged and used to detect SNPs between the two parental lines (Table 2). As a result, a total of 674,521 SNPs were found throughout nine chromosomes, with an average of 1 SNP per 662.5-bp interval. The highest density of SNPs was found on chromosome C03, with a SNP per 541 bp, while the lowest density was on chromosome C05, with one SNP per 818.9 bp. Analysis of the distribution of SNPs per 100 kb along the nine chromosomes revealed areas of high and low SNP density on each chromosome (Figure 1).

**Table 2:** **Summary of SNPs detected from** ***B. oleracea*** **whole-genome resequencing data and development of dCAPS markers for validation**

**Ch.**	**Number of SNPs (average bp per SNP)**						**Validation**		
	**Ref vs. C1184**		**Ref vs. C1234**		**C1184 vs. C1234**		**Amplified/Designed**	**Polymorphic (h)** ^**a**^	**%** ^**b**^
**C01**	**122,191**	**(358.2)**	**114,778**	**(381.3)**	**66,197**	**(661.1)**	**31 / 35**	**20 (4)**	**64.5%**
**C02**	**149,730**	**(353.2)**	**161,246**	**(328.0)**	**74,741**	**(707.6)**	**14 / 17**	**10 (1)**	**71.4%**
**C03**	**196,150**	**(331.3)**	**205,306**	**(316.5)**	**120,115**	**(541.0)**	**13 / 22**	**11 (1)**	**84.6%**
**C04**	**136,815**	**(392.6)**	**132,144**	**(406.5)**	**86,999**	**(617.5)**	**14 / 20**	**8 (4)**	**57.1%**
**C05**	**130,557**	**(359.2)**	**132,887**	**(353.0)**	**57,417**	**(818.9)**	**15 / 18**	**14 (2)**	**93.3%**
**C06**	**87,712**	**(454.1)**	**102,422**	**(388.8)**	**63,017**	**(631.9)**	**28 / 34**	**16 (4)**	**57.1%**
**C07**	**119,275**	**(405.5)**	**128,978**	**(375.0)**	**69,905**	**(691.9)**	**8 / 15**	**5 (1)**	**62.5%**
**C08**	**108,586**	**(384.6)**	**113,956**	**(366.4)**	**68,361**	**(610.9)**	**21 / 26**	**18 (3)**	**85.7%**
**C09**	**147,866**	**(369.8)**	**149,581**	**(365.6)**	**67,768**	**(806.9)**	**23 / 35**	**15 (6)**	**65.2%**
**Total**	**1,198,882**	**(372.8)**	**1,241,298**	**(360.0)**	**674,521**	**(662.5)**	**167 / 222**	**117 (26)**	**70.1%**

^a^h is the number of markers that showed heterozygous results.

^b^Percentage of the total amplified dCAPS markers that were polymorphic.

### Development of dCAPS markers and construction of genetic map

We used the SNPs between C1184 and C1234 for development of dCAPS markers. Based on the physical positions of all markers used in a previous genetic map for *B. oleracea* [21], new dCAPS markers were designed for the regions of low marker density. Among 167 markers amplified, 117 (70.1%) were polymorphic between the two parental lines (Table 2 and Additional file 1: Table S1). Among the 117 polymorphic markers, 26 showed heterozygosity in one of parental lines (Table 2). We used 87 of these polymorphic dCAPS markers for genotyping of each individual in the F_2_ population (Additional file 1: Table S1). Additionally, 16 other types of polymorphic markers including five EST-based dCAPS markers, five MIP markers, three IBP markers, two genomic SSR markers, and one INDEL marker were also genotyped with the same population. Among 103 newly analyzed markers, 25 markers showed a segregation pattern distorted from the 1:2:1 Mendelian ratio in the F_2_ population, based on chi-square goodness of fit at the 0.05 probability level (Additional file 2: Table S2). There were six segregation distortion regions (SDRs) in the previous map [21], and all dCAPS markers designed from the SDRs of C01 and C05 showed the same distortion ratio.

**Figure 2:**
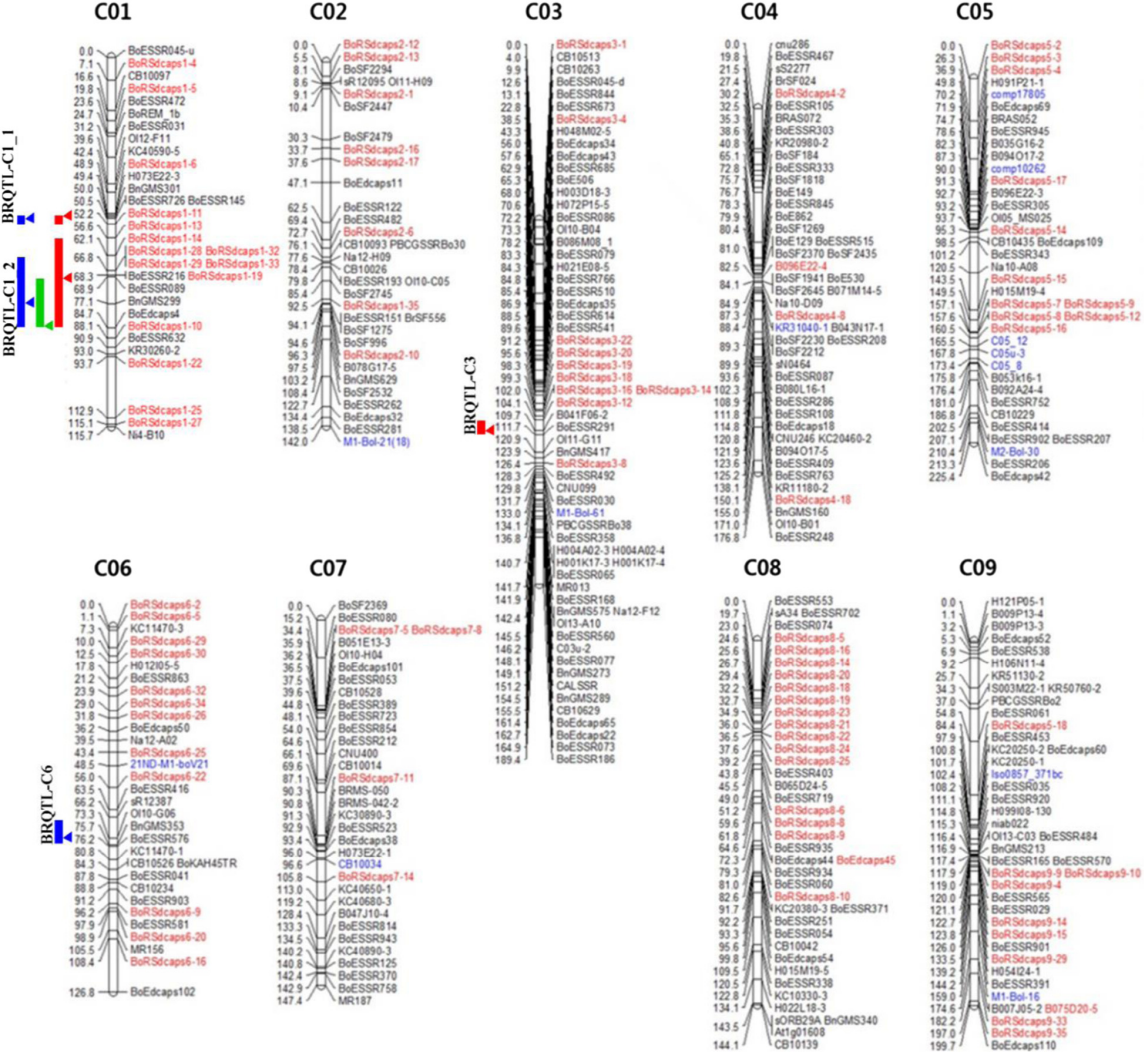
**Genetic linkage map of cabbage constructed using 368 markers.** Markers in red are newly developed dCAPS markers and markers in blue are EST-based dCAPs, MIP, IBP, genomic SSR, and INDEL markers. QTLs identified in inoculation tests in 2012, 2013, and 2014 are shown as red, green, and blue bars, respectively. The position of the peak LOD score in each QTL is indicated by an arrowhead.

The 103 novel polymorphic marker loci (Additional files 1 and 2: Tables S1 and S2) were added to the previous 265 markers [21] to develop a higher density genetic map. All 368 markers were placed on the map, and a linkage map was generated with nine linkage groups (LGs) in which each LG had more than 32 markers (Figure 2, Table 3). The improved *B. oleracea* genetic map spanned 1,467.3 cM, which is 135.4 cM more than the previous map, and the average distance between neighboring loci was reduced to 3.88 from 5.02 cM. Most of the new dCAPS markers were mapped to the originally estimated position of each chromosome sequence. The exceptions included BoRSdcaps1-35, which was designed on chromosome C01 but mapped to chromosome C02, and BoRSdcaps5-18, designed on chromosome C05 but mapped to chromosome C09.

**Table 3:** **Distribution of molecular markers on the cabbage genetic map**

**Marker type**		**C01**	**C02**	**C03**	**C04**	**C05**	**C06**	**C07**	**C08**	**C09**	**Total**
**This study**	**dCAPS**	**15**	**8**	**10**	**3**	**11**	**12**	**4**	**15**	**9**	**87**
	**Other markers** ^**a**^	**0**	**1**	**1**	**2**	**6**	**1**	**1**	**1**	**3**	**16**
**Previous study**		**18**	**25**	**52**	**43**	**23**	**19**	**29**	**25**	**31**	**265**
**Total**		**33**	**34**	**63**	**48**	**40**	**32**	**34**	**41**	**43**	**368**
**Length (cM)**		**115.7**	**142.0**	**189.4**	**176.8**	**225.4**	**126.8**	**147.4**	**144.1**	**199.7**	**1467.3**
**Average interval (cM)**		**3.51**	**4.18**	**3.01**	**3.68**	**5.64**	**3.96**	**4.34**	**3.51**	**4.64**	**3.88**

^a^Including MITE insertion polymorphism, EST-based dCAPS, Intron based polymorphism, genomic SSR, and INDEL markers.

### Black rot resistance assays and QTL analysis

We performed three independent inoculation trials over three years. The final disease index for F_2_ plants was determined by calculating the average value of the black rot disease indices for 10 ~ 15 F_2:3_ progeny plants for each trial. Although all three inoculation tests were performed under the same conditions, the disease symptoms for each test were not consistent and tended to become more severe in later years (Additional file 3: Figure S1), possibly due to differences in plant growth or storage term for the F_3_ seeds or to weather differences between years.

QTL analyses were performed for each of three trials. We detected significant QTLs, based on higher LOD scores than the thresholds calculated in the permutation tests; LOD threshold values for the tests in 2012, 2013, and 2014 were 3.063, 2.912, and 2.906, respectively. In the first test performed in 2012, there were three significant QTL regions: BRQTL-C1_1 and BRQTL-C1_2 on chromosome C01, and BRQTL-C3 on chromosome C03 (Figure 2). Among these, BRQTL-C1_2 had the highest LOD score, additive effect, and variance explained (Table 4, Figure 2). The second test identified only a single QTL, which was included within BRQTL-C1_2 detected in the 2012 test, although this QTL had smallest LOD score among all QTLs identified in the three tests. The last test, carried out in 2014, identified BRQTL-C1_1 and 2 as well as a novel QTL in chromosome 6, BRQTL-C6. BRQTL-C1_2 in the 2014 test was identified as a smaller region than in 2012, but had the highest LOD score among all QTLs and accounted for 27.3% of the variation.

**Table 4:** **QTLs identified for resistance to** ***Xcc*** **KACC 10377**

**Inoculation test**	**QTL name**	**Linkage group**	**Marker interval (cM)**	**Marker nearest to peak in LOD score**	**LOD** ^**a**^	**Additive effect** ^**b**^	**Variance explained (%)** ^**c**^
**1st test (2012)**	**BRQTL-C1_1**	**C1**	**H073E22-3 - BoRSdcaps1-11 (2.8 cM)**	**BnGMS301**	**3.871**	**−0.714**	**17.8**
	**BRQTL-C1_2**	**C1**	**BoRSdcaps1-13 - BoEdcaps4 (28.1 cM)**	**BoESSR089**	**4.720**	**−0.697**	**21.2**
	**BRQTL-C3**	**C3**	**BoRSdcaps3-12 - BoESSR291 (7.6 cM)**	**B041F06-2**	**3.834**	**−0.661**	**17.6**
**2nd test (2013)**	**BRQTL-C1_2**	**C1**	**BoESSR089 - BoEdcaps4 (15.8 cM)**	**BoEdcaps4**	**3.051**	**−0.602**	**15.1**
**3rd test (2014)**	**BRQTL-C1_1**	**C1**	**H073E22-3 - BoRSdcaps1-11 (2.8 cM)**	**BoESSR726, BoESSR145**	**3.881**	**−0.912**	**19.8**
	**BRQTL-C1_2**	**C1**	**BoRSdcaps1-14 - BoEdcaps4 (22.6 cM)**	**BnGMS299**	**5.619**	**−0.987**	**27.3**
	**BRQTL-C6**	**C6**	**sR12387 - BnGMS353 (9.5 cM)**	**Ol10-G06**	**3.847**	**−0.868**	**19.6**

Shown are position of the QTL on the map, LOD scores, additive and dominant effects, and percentage of variance explained.

^a^Peak LOD score of the QTL.

^b^Additive or dominant effect of C1234 allele.

^c^Percentage of variance explained at the peak of QTL.

### NBS-encoding genes in QTL regions

In most plants, disease resistance-related genes (R genes) encode proteins containing nucleotide binding sites (NBS) and a series of leucine-rich repeats (LRRs), termed NBS-LRR proteins. NBS-LRR proteins recognize and correspond to pathogen avirulence factors, and lead to defense responses and hypersensitive reactions [22]. Hence, we compared our genetic map to the pseudo-chromosome sequences [3] and searched for NBS-LRR genes within the QTL regions (Table 5). BRQTL-C1_1 was found between markers H073E22-3 and BoRSdcaps1-11, and BRQTL-C1_2 was between BoRSdcaps1-13 and BoEdcaps4 (Table 4). We identified eight NBS-LRR-encoding genes between H073E22-3 and BoEdcaps4 showing BRQTL-C1_1 and BRQTL-C1_2 QTLs. Seven NBS-LRR type R genes were detected within 1 Mb of the BoESSR291 marker, which is located near the BRQTL-C3 region. BRQTL-C6 contained five NBS-LRR type R genes.

**Table 5:** **NBS-LRR-encoding genes in black rot resistance QTL regions identified for** ***B*** **.** ***oleracea*** **in this study, and syntenic orthologs in closely related species**

**QTL region in** ***B. oleracea***	**Genes in** ***B. oleracea*** **(Parkin et al. 2014 [** 14 **])**	**Orthologs in** ***B. rapa***		**Syntenic genes in** ***A. thaliana***
		**Gene ID** ^**b**^	**Position in** ***B. rapa***	
**BRQTL-C1_1**	**Bo1g056920**	**Bra034079**	**A01: 25,091,903 - 25,095,843**	
**C01: 14,884,502 - 16,579,946**				
**BRQTL-C1_2**	**Bo1g057060/070**	**Bra039560**	**A01: 11,678,267 - 11,687,802**	**AT4G14380**
**C01: 18,227,386 – 37,119,290**				
	**Bo1g086130**	**Bra013691**	**A01: 7,172,559 - 7,175,366**	**AT4G23440**
	**Bo1g087610**	**Bra038144**	**Scaffold000140**	**AT1G57850**
	**Bo1g091560**			
	**Bo1g094680/710** ^**a**^	**Bra031456/455** ^**a**^	**A01: 17,128,737 – 17,140,522**	**AT1G61100/105** ^**a**^
	**Bo1g103860**			
**BRQTL-C3**	**Bo3g060060/070/080/100/110/130/140** ^**a**^	**Bra001160/161/162** ^**a**^	**A03: 15,040,407 - 15,054,981**	
**C03: 19,714,632 - 22,846,644**				
**BRQTL-C6**	**Bo6g025490**	**Bra004192**	**A07: 20,618,348 - 20,627,341**	**AT1G66840**
**C06: 7,423,787 - 10,466,894**	**Bo6g031330/350/360/380**	**Bra003997**	**A07: 19,462,054 - 19,467,133**	**AT1G69550**

^a^Tandemly arrayed genes.

^b^Gene ID in BRAD database (http://brassicadb.org/).

We compared the sequences of these 21 candidate R genes against the *Brassica* Database (BRAD; http://brassicadb.org/) [23]. All 21 sequences showed similarity to disease resistance proteins, of which 19 and 11 sequences had syntenic genes in *B. rapa* and *A. thaliana*, respectively. According to the gene annotation, two candidate disease resistance genes (Bo1g094680 and Bo1g094710) in BRQTL-C1, and seven genes in BRQTL-C3 were found as gene clusters (Table 5).

**Figure 3:**
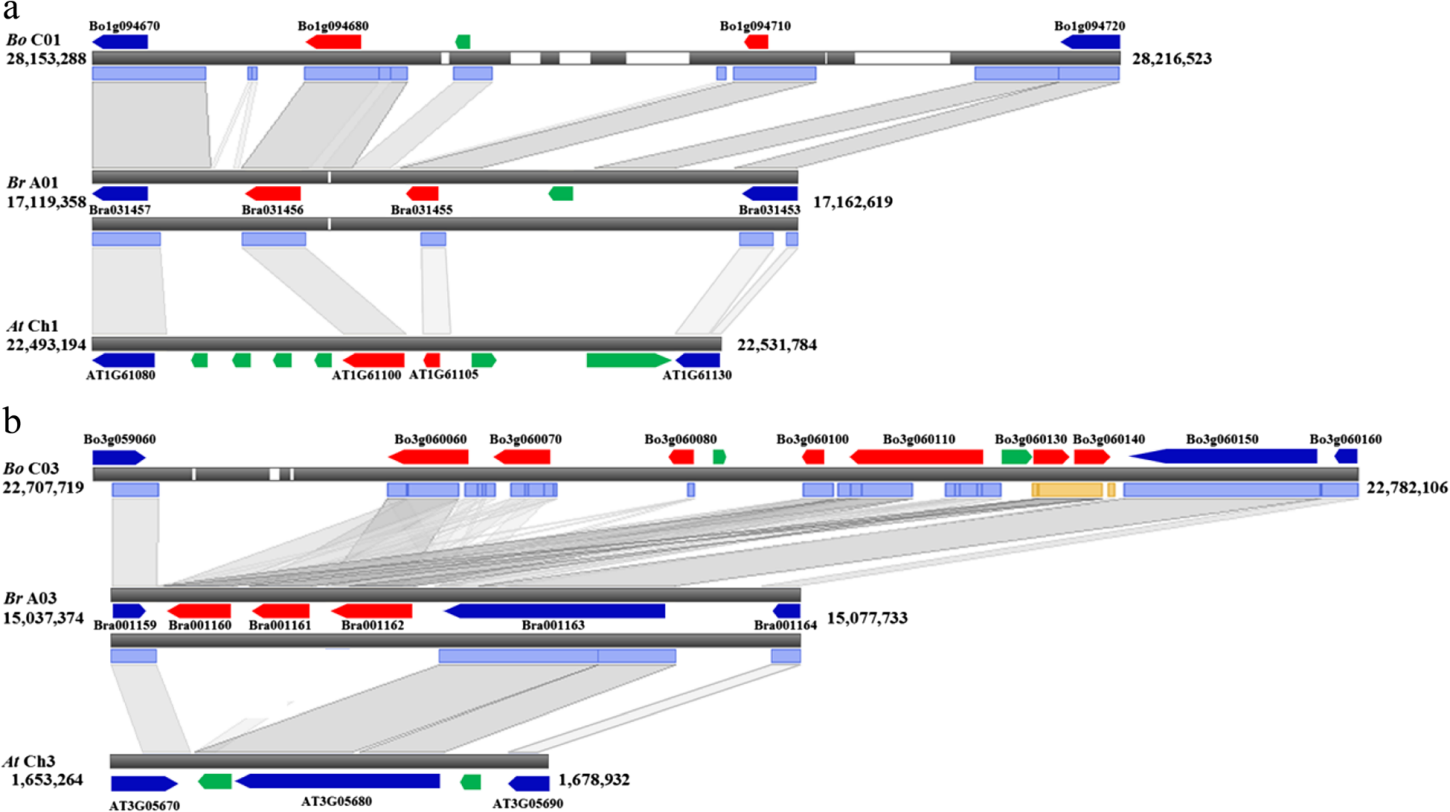
**Syntenic relationships among crucifer species of QTL regions containing genes encoding NBS-LRR proteins.** Black bars represent the chromosomal blocks and white regions are N-gaps. Red and blue indicate genes for which orthologous genes were found in relative species, with those encoding NBS-LRR genes in red and non-NBS-LRR genes in blue. Green denotes genes that were annotated only in one species; Bo-*Brassica oleracea*, Br-*Brassica rapa*, and At-*Arabidopsis thaliana*. **(a)** Syntenic regions that include disease resistance genes **(b)** Syntenic regions with different numbers of NBS-LRR-encoding genes clustered in *B. oleracea* and *B. rapa.*

Seven of nine NBS-LRR genes in BRQTL-C1 were syntenic with the R genes in the counterpart regions of chromosome A01 in *B. rapa* (Table 5). Orthologous genes of two NBS-LRR genes, Bo1g094680 and Bo1G094710, located within a 63-Kb portion of BRQTL-C1 appeared as tandem array at the counterpart syntenic region in *B. rapa* and *A. thaliana* (Figure 3a). All NBS-LRR genes in BRQTL-C6 also showed highly conserved syntenic relationships with counterpart regions in *B. rapa* and *A. thaliana*. However, a 72-Kb region near BRQTL-C3 contained a cluster of seven NBS-LRR genes, whereas the syntenic region in *B. rapa* contained a cluster of only three such genes, and the corresponding syntenic region in *A. thaliana* did not have any R genes (Figure 3b).

## Discussion

### Frequency and utility of SNPs revealed by whole-genome resequencing

An appropriate reference sequence allows whole-genome sequence data from individuals to be aligned, and thus resequencing can be used to detect genetic variation between different samples as high-confidence sequence differences [24]. Accordingly, we performed whole-genome resequencing for two cabbage lines to detect genome-wide SNPs for marker development to construct a high-density genetic map. There are two available draft genome sequences for *B. oleracea*; although the total assembled sequence of Liu et al. (539.9 Mb) [2] is larger than that of Parkin et al. (488.6 Mb) [3], the size of the nine pseudo-chromosomes of the latter (446.9 Mb) is larger than that of the former (388.8 Mb). Therefore, we chose the genome sequence of Parkin et al. [3] as a reference for this research because of its advantage for sequence-guided SNP marker development.

Approximately 80% of our newly generated PE sequence reads were successfully aligned to the reference genome. Almost 1.2 million SNPs were found in both lines compared to the reference, and 670,000 SNPs were found between C1184 and C1234. The number and density of SNPs between both parental lines were much lower than those detected by comparison with the reference. This could be related to the fact that the plant material used for the reference genome sequencing was kale-like *B. oleracea* [3], whereas the two parental lines used in this work were typical cabbages. In addition, we detected fewer SNPs between these lines than the 1.42 million SNPs (averaging one SNP in every 360 bp) previously reported between two other cabbages [25]. Our plant materials have been used as elite breeding resources by a Korean company, and thus the genetic relationship between these two parental inbred lines is likely much closer than to the reference accession or the relationship between the two cabbages used by Liu et al. [25]; this close relationship likely underlies the relatively low number of SNPs we identified.

Among 167 dCAPS markers that successfully produced PCR products, 70.1% showed polymorphism. This rate was much higher than that of EST-derived dCAPS markers, in which the polymorphic rate was 58.44% when evaluated with low-sequencing depth 454 RNA-seq reads [21]. Paired-end read data generated by Illumina sequencing could allow more accurate alignment of raw reads compared to single reads from 454 RNA-seq, and thus the SNP calling process would also become more precise. The 29.9% of dCAPS markers showing no polymorphism might reflect false mapping of reads to paralogous regions, as there is high sequence similarity between the triplicated genomes and among recently duplicated chromosome segments [26-28]. This could also be the reason two dCAPS markers, BoRSdcaps1-35 and BoRSdcaps5-18, were mapped to unexpected chromosomes.

Collectively, our results demonstrate that whole-genome resequencing data generated by NGS techniques can be highly useful for large-scale discovery of SNPs and development of SNP-based molecular markers. Further study will enable high-throughput genotyping with SNPs detected here.

### Improvement of the genetic map between cabbage breeding lines

By obtaining large numbers of reliable SNPs and utilizing them for development of DNA markers, we were able to improve the genetic map of cabbage. The genetic map now spans a total 1,467.3 cM after our addition of SNP markers developed for the relatively large gaps (greater than 20 cM) in the previous map [21]. Consequently, the 12 gaps in the previous genetic map are now reduced to 6 gaps and the average interval is smaller than before. The 368 markers used for the improved genetic map are promising for general cabbage breeding purposes because the map was built using a mapping population between two elite breeding lines with narrow genetic diversity. By contrast, most of previous genetic map was built using mapping populations derived between lines with wide genetic diversity for academic purposes, for example a cross between double-haploid (DH) lines derived from other subspecies [3,29] or DH lines selected based on simple morphological differences [20]. Therefore, the genetic map in this study will be helpful for molecular breeding associated not only with black rot resistance but also with many other important agricultural traits.

### QTL mapping of black rot resistance

We identified four QTL regions that could contribute additively to resistance. The BRQTL-C1_2 QTL region was detected repeatedly in the three independent inoculation tests, had the highest LOD values and also accounted for the highest percentage of the variation in all tests. Accordingly, BRQTL-C1_2 is a strong candidate to be a major QTL for black rot resistance. BRQTL-C1_1, BRQTL-C3, and BRQTL-C6 seem to be minor QTLs, which could be influenced by plant conditions and environmental factors. Although two QTLs identified in chromosome C1 included SDRs, we retained all distorted markers for QTL analysis because distorted markers can also be helpful for QTL mapping when they are addressed properly [30].

The positions of our black rot resistance QTLs did not coincide with those of the 14 previously reported QTLs [4,5,15,16]. This lack of overlap is probably due to differences in disease resistance sources or inocula used. Some studies did not describe the races used [15,16], while some [4,5] used *Xcc* race 1. The exact race used in this study has not been classified yet. Integrated and standardized protocols for black rot disease races and testing would facilitate further research. However, even though the same *Xcc* race was used in our three inoculation tests, disease indices for same F_2_ lineages were not consistent year to year and thus different QTLs were detected between tests. Resistance to *Xcc* has been reported to vary depending on accessions of *B. oleracea* and pathogen races [31,32]. Further, the resistance is likely also affected by complex polygenic control under different environmental conditions. Regardless of the race used (if the same as in previous studies or not), the different QTLs detected here should represent new regions.

### Candidate genes for black rot resistance

The genomes of *B. oleracea*, *B. rapa*, and *A. thaliana* share a set of 24 conserved syntenic blocks, A to X, that can be identified among the ancestral karyotype [33]. The complete *B. oleracea* draft genome also demonstrates generally strong conservation with *B. rapa* in large segments at the pseudo-molecule level [2,3]. Comparative analysis revealed the presence of conserved R gene orthologs at the syntenic counterparts in *B. oleracea*, *B. rapa* and *A. thaliana*. In particular, the BRQTL-C1 region of C1 in *B. oleracea* showed large-scale conservation with A01 in *B. rapa*. Our analysis demonstrated that Bra038144, found in unanchored scaffold000140 of the *B. rapa* genome, is an ortholog of Bo1g087610 in *B. oleracea* (Table 5). Based on our finding that AT1G57850, the corresponding orthologous gene in *A. thaliana,* was also located in a syntenic region, the unanchored *B. rapa* scaffold000140 is likely derived from chromosome A01.

In plant genomes, hundreds of NBS-LRR genes are distributed as single genes or in tandem arrays as gene clusters, which arise from tandem gene duplications or homologous recombination and homogenization [34,35]. We detected 21 R genes in the four QTL regions, of which 9 were in gene clusters (Table 5). Most of the R genes showed conserved syntenic relationships in *Brassica* and *Arabidopsis* (Figure 3a). However, near BRQTL-C3 were NBS-LRR gene clusters that appear to be unique to the *Brassica* lineage (Figure 3b). This result implied that three-R-gene clusters arose by insertion in the *Brassica* lineage at *Br*A03 and subsequently amplified to a seven-R-gene cluster in *B. oleracea* over the 4.6 million years after divergence of the *Brassica* species [2].

Although genomes of *Brassica*-lineage species underwent whole-genome triplication events, the number of resistance genes was not proportionally increased in the *Brassica* genome [27,36]. Around 150 ~ 200 R genes were reported in the *A. thaliana* genome [2,35,37], and 206 [2] ~ 244 (http://brassicadb.org/) and 157 genes [2] were annotated as R genes in *B. rapa* and *B. oleracea* genomes, respectively. The 21 NBS-LRR genes found in the four QTL regions are proportionally higher density compared to other chromosomal regions, supporting the idea that some of these NBS-LRRs could be candidate to control black rot resistance in *B. oleracea*. Further analysis to reveal the function of these genes will be necessary for identification of the major resistance genes for *Xcc*.

## Conclusion

We performed whole-genome resequencing of two cabbage inbred lines that are parental lines for black rot disease resistance and breeding lines with elite agricultural traits. Based on genome-wide SNP detection and validation with dCAPS markers, we report 670,000 SNPs with 70% accuracy between the parental lines. By combining SNP-based markers into the previous genetic map, we improved the genetic map and identified four QTL regions that contained 21 candidate R genes. We thus demonstrated that whole-genome resequencing can successfully be applied for detection of large-scale SNPs, development of molecular markers, and ultimately construction of a high-density genetic map for QTL analysis and marker-assisted breeding of *B. oleracea*.

## Methods

### Plant materials and whole-genome resequencing

Two cabbage (*Brassica oleracea L*. var. *capitata*) inbred lines, C1184 and C1234, were selected as parents to develop a mapping population. The two lines show different responses to black rot disease; C1184 is susceptible to *X. campestris* pv. *campestris* (*Xcc*), whereas C1234 is resistant. The mapping population consisted of 97 F_2_ plants generated by crossing between C1184 and C1234, as described previously [21]. Furthermore, the 97 F_2_ plants were vernalized and self-pollinated to produce seeds of F_3_ progenies for inoculation tests. All plant materials examined in this study were obtained from Joeun Seeds Co. (Chungcheongbuk-Do, Korea).

Genomic DNAs were extracted from approximately 5 g samples of young leaves from the cabbage parental lines, following the modified cetyltrimethylammonium bromide (CTAB) protocol [38]. The quality and quantity of the DNA were examined using a NanoDrop ND-1000 (NanoDrop Technologies, Inc., USA). More than 5 μg extracted DNA was randomly sheared and quantified using DNA 1000 kit (Agilent Technologies, Inc., USA) according to the manufacturer’s protocol. Sequencing with constructed shotgun libraries of C1184 and C1234 was performed by Illumina Hi-seq 2000. Fragmentation, library construction, and sequencing were carried out by the National Instrumentation Center for Environmental Management (NICEM; Seoul, Korea).

### SNP discovery and dCAPS marker design

Overall process of SNP discovery was performed by following the framework described by DePristo et al. [39]. Briefly, Illumina paired reads from the parental lines were aligned to the reference sequence of *B. oleracea* [3] using Bowtie2 program [40]. Then, read grouping and removal of PCR duplicates were carried out using Picard (http://picard.sourceforge.net). Misalignments caused by INDELs were corrected by local re-alignment using Genome Analysis Toolkit (GATK) and candidate SNPs were called using Variant Caller, a utility in GATK [41]. To filter variants and avoid false positives, candidate SNPs exhibiting any of the following characteristics were removed: (1) mapping quality score lower than 4; (2) quality less than 30; (3) less than 10× or more than 45× mapping depth.

Initially, SNPs of C1184 and C1234 relative to the reference genome were called separately. All of the identified SNP positions from both parental lines were then merged and compared to each other, and promising SNPs for this research between C1184 and C1234 were identified. The selected SNPs were used to develop dCAPS markers using the dCAPS Finder 2.0 program (http://helix.wustl.edu/dcaps) for design of nearly-matched primers including SNP positions. After designing such mismatched primers for each SNP, the opposite primers were designed using the Primer3 program (http://primer3.wi.mit.edu/). All primers were synthesized by Macrogen (Seoul, Korea).

### Molecular marker analysis

The newly developed dCAPS markers were validated by examining polymorphisms between the two parental lines C1184 and C1234. Additional expressed sequence tag (EST)-based dCAPS, intron-based polymorphic (IBP), genomic SSR, and INDEL markers that were not included in the previous genetic map [21] were also analyzed in this study. Furthermore, five polymorphic markers based on miniature inverted transposable element (MITE) insertion polymorphism (MIP) [42,43] were also used for genotyping the F_2_ population.

PCR amplifications were performed in a total volume of 25 μL containing 20 ng genomic DNA template, 1 × PCR buffer, 20 pM each primer set, 0.2 mM each dNTP, 1 U Taq DNA polymerase (VIVAGEN, Korea). The amplicons of dCAPS markers were mixed with 3 U appropriate restriction enzymes (New England Biolabs, USA), the corresponding 1 × buffer, and 1 × BSA when necessary, then incubated at 37°C for more than three hours. The digested fragments of dCAPS markers and amplicons of other markers stained by ethidium bromide were visualized on a UV trans-illuminator after electrophoresis using 9% non-denaturing polyacrylamide gels or 1% agarose gels depending on fragment size.

### Inoculation test

*Xanthomonas campestris pv. Campestris* KACC 10366, obtained from the Korean Agricultural Culture Collection (KACC; Suwon, Korea) were used for the inoculation tests. Inoculum of the bacterium was scraped and cultured on tryptic soy agar (TSA) plates at 30°C for 48 h. Cultured bacteria were harvested using a spreader and diluted with distilled water to 0.125 OD at 600 nm to prepare bacterial suspension for inoculation.

**Figure 4:**
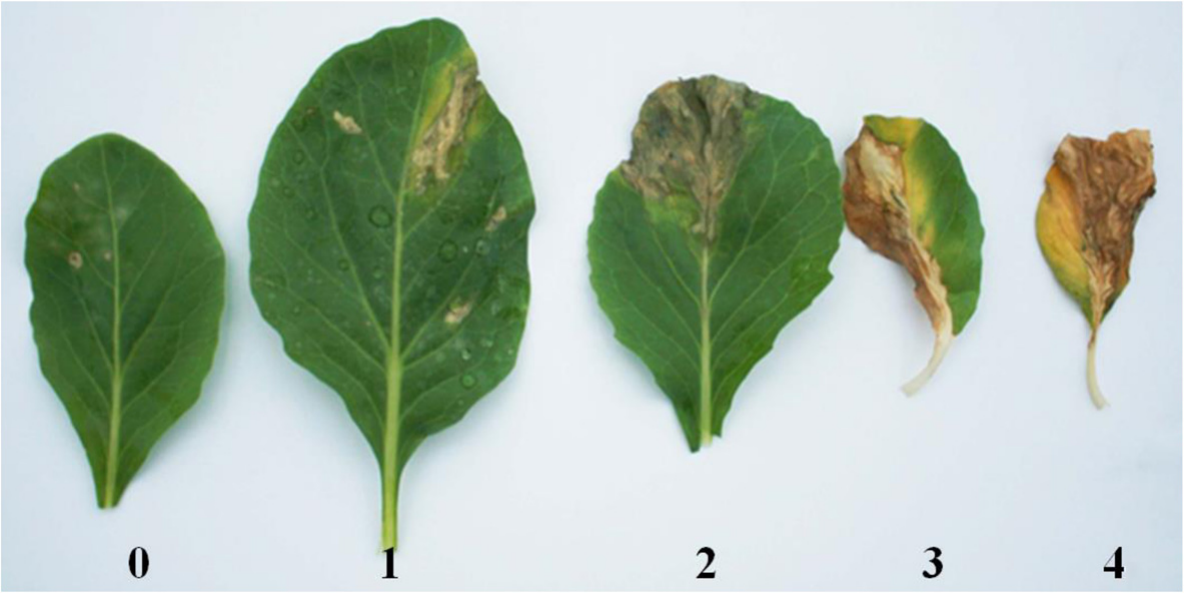
**Representative black rot disease symptoms on leaves of** ***B. oleracea*** **after spraying with** ***Xcc*** **suspension.** Disease indices are: **(0)** less than 15%, **(1)** 15-30%, **(2)** 30-55%, **(3)** 55-75%, **(4)** more than 75% leaf area showing black rot symptoms.

Inoculation tests, carried out in 2012, 2013, and 2014 under the same conditions at the Korea Research Institute of Chemical Technology (Dae-jeon, Korea), were performed with 10 ~ 15 F_3_ plants of each individual F_2_ plants selected for genotyping analysis. The F_3_ seeds were sown and grown on 5 × 8 plastic pots for 20 d in a greenhouse. Afterwards, 20-d-old plants, usually at a stage with two sufficiently developed true leaves, were inoculated by spraying bacterial suspension until adaxial and abaxial surfaces of leaves were sufficiently wet. Each plastic pot (40 plants) received 80 mL bacterial suspension, and the inoculated plants were moved into a dew chamber with the temperature set at 28°C. After 48 h incubation, all plants were transferred to a room maintained at 25°C and 80% humidity for further 7 d incubation with 12 h light/day, and disease symptoms on two inoculated leaves per each plant were surveyed. The severity of the black rot symptoms were recorded based on infected leaf area, with the following disease indices: (0) less than 15%, (1) 15-30%, (2) 30-55%, (3) 55-75%, (4) more than 75% leaf area showing black rot symptoms (Figure 4).

### Map construction and QTL analysis

A total of 103 polymorphic markers were genotyped in the F_2_ population, and the resulting scores were integrated into genotyping data used for a previous genetic map [21]. Linkage analysis and map construction were performed using JoinMap version 4.1 with the same parameters as in the previous study [21]. The Kosambi mapping function was used to convert recombination frequencies into genetic distances.

A disease index for each F_2_ individual was calculated as the mean grade of 10 ~ 15 F_3_ seedlings. QTLs for *Xcc* resistance were evaluated using composite interval mapping (CIM) analysis with QGene program. CIM was performed with LOD (logarithm of odds) threshold values that were estimated using 1,000 permutation tests at 5% significance with 0.5-cM scan intervals.

## Additional files

Additional file 1: Table S1. Description of polymorphic markers between C1184 and C1234 used in this study. Additional file 2: Table S2. Results of the chi-square goodness-of-fit tests for the observed segregation ratios with the genotyped markers among F_2_ plants. Additional file 3: Figure S1. Disease index distribution of F_2_ population, evaluated by average scores from inoculated F_3_ plants.

## Abbreviations

QTL: Quantitative trait loci
SNP: Single nucleotide polymorphisms
Xcc: *Xanthomonas campestris* pv. *campestris*
SSR: Simple sequence repeat
NGS: Next-generation sequencing
NBS: Nucleotide binding sites
LRRs: Leucine-rich repeats
GATK: Genome analysis toolkit
EST: Expressed sequence tag
dCAPS: Derived cleaved amplified polymorphic sequences
IBP: Intron-based polymorphic
MITE: Miniature inverted transposable element
MIP: MITE insertion polymorphism
CIM: Composite interval mapping
MAS: Marker-assisted selection
CTAB: Cetyltrimethylammonium bromide
