# Supplementary material for: Genome-wide SNP identification and QTL mapping for black rot resistance in cabbage
Source: BMC Plant Biol. 2015 Feb 3;15:32. doi: 10.1186/s12870-015-0424-6 (PMC4323122; doi:10.1186/s12870-015-0424-6)

Additional file 3 Figure S1. Disease index distribution of F_2 population, evaluated by average scores resulted from inoculated F_3 plants

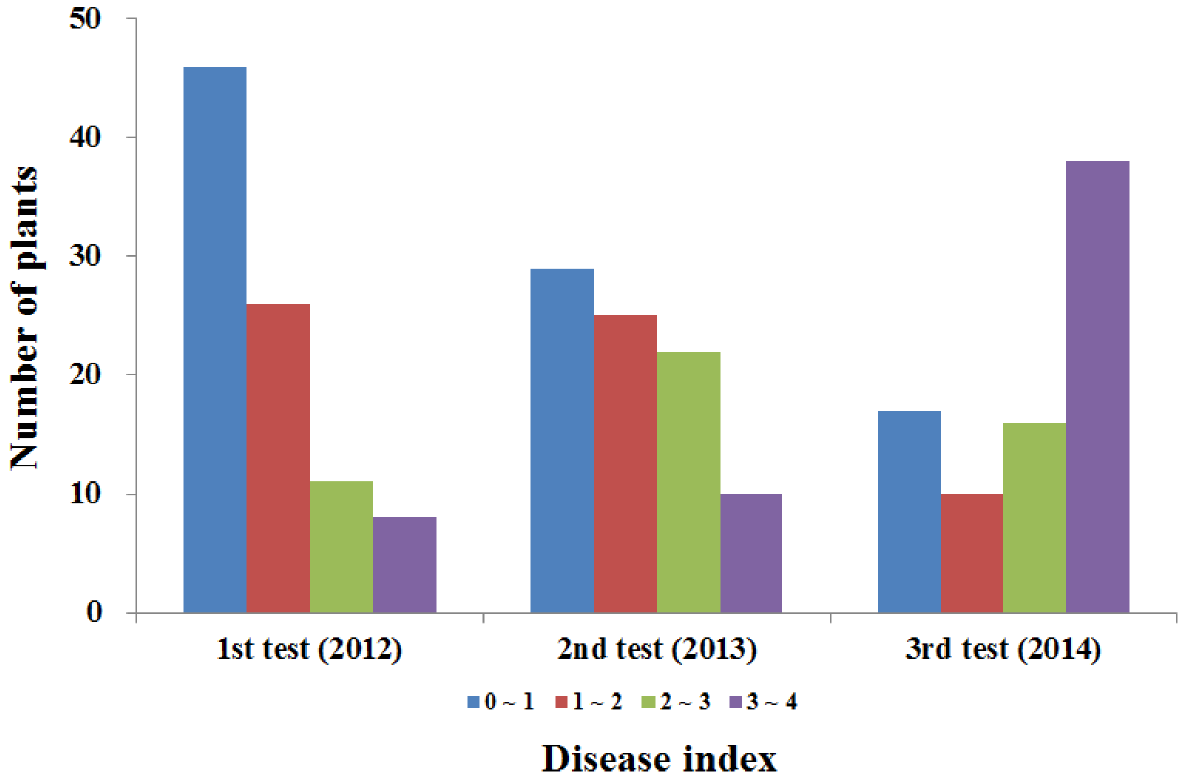

Supplement: Additional file 3: Figure S1. — Disease index distribution of F2 population, evaluated by average scores from inoculated F3 plants. [file 12870_2015_424_MOESM3_ESM.pdf]
